# Comprehensive Review on Association of Bipolar Disorder and Substance Abuse: Dual Diagnosis and Treatment Approaches

**DOI:** 10.7759/cureus.86363

**Published:** 2025-06-19

**Authors:** Felicia T Bonner-Reid, Arzoo Dar, Annu Zerin, Anushka Prakash Mishra, Claudia Lollobattista, Niketa Narasimhan, Smit Desai, Satneet Singh

**Affiliations:** 1 Medicine and Surgery, University of Medical Sciences of Granma (Celia Sánchez Manduley), Manzanillo, CUB; 2 Medicine, Hull York Medical School, York, GBR; 3 Department of Medicine, All India Institute of Medical Sciences, Bhubaneswar, IND; 4 Department of Medicine, Era's Lucknow Medical College and Hospital, Lucknow, IND; 5 Department of Psychology, Università Vita-Salute San Raffaele, Milan, ITA; 6 Department of Medicine, Miami University, Miami, USA; 7 Department of Medicine, Gujarat Medical Education and Research Society (GMERS) Medical College and Hospital, Gandhinagar, IND; 8 Psychiatry, Southern Health NHS Trust, Fareham, GBR

**Keywords:** bipolar disorder, comorbidity, dual diagnosis, psychotherapy approaches, substance abuse disorder

## Abstract

Bipolar disorder (BD) and substance use disorder (SUD) usually arise in conjunction, leading to worse outcomes than when either condition exists independently. This co-occurrence is associated with a greater number of severe symptoms, longer illness duration, higher suicide rates, and increased overall health problems. The growing number of individuals affected by both BD and SUD has sparked interest in understanding their connection and finding effective treatment strategies. This research paper provides a comprehensive overview of the high rates of SUD among people with BD. It explores certain determinant factors such as genetic polymorphism, neurobiological implications, and psychosocial and environmental factors that contribute to this association. It also discusses the challenges in diagnosing both conditions, given their overlapping symptomatology, and the importance of recognizing dual diagnosis in clinical settings. Treatment options, including medications, psychotherapy, and integrated care models, are examined, focusing on tailored approaches for patients. The review also touches on the stigma surrounding dual diagnosis, which can affect treatment engagement. Lastly, it highlights the promise of innovative therapies and emphasizes the need for more research to improve detection, medical care, and attention.

## Introduction and background

Persistent emotional, mental, or behavioral alterations that considerably affect one’s capacity to function and engage in day-to-day life could reflect an underlying affective disorder [1]. Depressive disorder and bipolar disorder (BD) are two main examples of affective disorder [1]. BD is a chronic and recurrent psychiatric condition marked by alternating episodes of mania, hypomania, and depression, affecting over 1% of the global population, regardless of ethnicity, nationality, or socioeconomic status [1-3]. BD is a leading cause of disability among young people, contributing to cognitive and functional impairments, as well as higher mortality, secondary to suicide [3]. Individuals with BD frequently experience both psychiatric and medical comorbidities, which can complicate accurate diagnosis [1]. This is especially true during depressive episodes, where BD may be mistaken for unipolar depression [1]. The combined prevalence of bipolar disorder types I and II is estimated at 1.6-2%, both of which significantly impact individuals' daily lives, relationships, and quality of life [2,3]. Furthermore, bipolar spectrum disorder prevalence rates range from 2.8 to 6.5% [3].

Substance use disorders (SUDs) involve the excessive and harmful use of substances like alcohol, drugs, or prescription medications, leading to impairment or distress [4]. Strong compulsion to use the addictive substance, struggles controlling use, and persistent use despite negative effects are typical features associated with SUDs [4,5]. SUDs are a major public health issue, contributing to morbidity, mortality, and healthcare costs [4,5]. Epidemiological studies have consistently shown that SUDs are common in individuals with personality disorders [6]. Lifetime estimates of alcohol abuse in BD range from 6% to 69%, with most studies reporting rates of 30% or higher [4,7]. Alcohol and cannabis are the most frequently abused substances, followed by cocaine and opioids [8]. Less favorable outcomes, which might include more frequent affective episodes, worse treatment adherence, lower quality of life, and a surge in suicidal thoughts, are linked to simultaneous SUDs in BD [8]. Individuals with bipolar disorder experience markedly elevated rates of substance use disorders, with lifetime prevalence estimates reaching at least 40%-a figure substantially higher than that observed in the general population [9-11]. Nearly 60% of people who have been hospitalized for manic or mixed episodes have been identified as having SUD at some point in their lives [12]. Co-occurring SUDs also correlate with negative outcomes such as suicide [13], suicide attempts [14,15], poor insight into the illness, and treatment non-adherence [16].

The term "dual diagnosis" is commonly used to describe individuals with two consecutive mental disorders, i.e., bipolar disorder and substance use disorder [17,18]. The interaction between BD and SUDs is significant, as each condition can exacerbate the other. For example, individuals with BD may use substances to self-medicate mood symptoms, while substance use can trigger or worsen mood episodes in BD [17,18]. The complexity of dual diagnosis leads to poorer outcomes, including more severe symptoms, higher hospitalization rates, increased suicide risk, and poorer treatment responses compared to those with a single disorder [18].

The focus of this review is to put forth an in-depth investigation of dual diagnosis, which is the coexistence of BD and SUDs. It explores the epidemiology, etiology, and clinical manifestations of this comorbidity, with a focus on the bidirectional relationship between BD and SUDs. Key topics include prevalence rates, demographic factors, and public health implications. Additionally, this review examines genetic, neurobiological, and psychosocial factors contributing to comorbidity, as well as challenges in diagnosis and treatment. It also provides a detailed analysis of pharmacological and psychotherapeutic interventions, integrated treatment models, and barriers to effective care. Ultimately, this review highlights the need for tailored, integrated approaches to improve patient outcomes, aiming to advance understanding and inform clinical practice in managing dual diagnosis.

## Review

Methodology 

This review adopts a comprehensive narrative approach to explore the association between BD and SUD, focusing specifically on dual diagnosis and associated treatment strategies. A comprehensive search was conducted across PubMed and Google Scholar to find relevant articles. We used several search terms in combinations such as "bipolar disorder," "substance use disorder," "dual diagnosis," "comorbidity," "pharmacotherapy," "psychotherapy," "integrated treatment," and "treatment approaches", among others. 

Articles that provided significant insights into epidemiology, pathophysiology, clinical manifestations, diagnostic challenges, and treatment interventions for BD and SUD comorbidity were included. Ethical approval and informed consent were not required as this work constitutes a literature review rather than primary research.

Epidemiology of dual diagnosis

An estimated 0.53% of the global population is affected by BD [19], with an approximated lifetime prevalence varying from 2.8 to 6.5% [3]. BD equally affects men and women and exhibits a bimodal peak incidence at ages 15-24 and 45-54 years [20]. In addition to cyclical mood disturbances, BD is commonly associated with drug misuse [21]. SUD is defined as a mental and behavioural illness characterised by the repeated, persistent use of psychoactive substances to seek pleasure or mitigate unpleasant withdrawal symptoms [22].

A systematic review concluded that approximately 42% of patients with SMI such as BD had comorbid alcohol use disorder (AUD) [23]. Data from a United States national survey suggested that BD I patients are at a six-fold increased lifetime risk of SUD [24]. A study found that the most commonly misused substance in BD was alcohol, followed by cannabis [25]. AUD rates in BD ranged from 19-58%, with a greater incidence in BD I than in BD II [26]. Heavy alcohol use has been found to perpetuate depressive symptoms, which may explain the higher suicide rates in dual-diagnosis patients [27]. Studies have found a higher prevalence of 49.3% for co-existing AUD and BD I versus 38.9% for AUD and BD II [28]. Historical literature suggests that BDI is substantially associated with SUD (p<0.05) [29].

The use of psychoactive substances is more commonly witnessed in younger adults, particularly males [30]. A positive family history of drug misuse is another risk factor. Offspring of parents with AUD may be at a twofold increased risk of this themselves, as demonstrated in a Danish cohort study [31]. The risk was particularly greater in females versus males, suggesting a disparity between the sexes. Socio-demographic factors such as unemployment, low household income, and lack of a partner may cause higher rates of BD; however, the evidence is not concrete [32]. In contrast, some studies have suggested that BD is more prevalent among those of higher socio-economic status or well-off occupations [33]. Similar demographic characteristics are seen in dual diagnosis, with higher incidence in younger adult males, with the majority being single, separated, or divorced [33]. Whether the SUD came first or the affective disorder is a question extensively studied and somewhat difficult to predict. However, it is widely accepted that substance misuse may either precipitate or be caused by BD.

Comorbid SUD and BD have been associated with early illness onset, greater morbidity due to increased symptom severity, and frequent hospitalizations, given the higher relapse risk [30,34]. Treatment failure due to interactions between misused substances and mood-stabilizing agents is a likely consequence that may also increase disease severity [7]. This puts a significant burden on healthcare systems, particularly in nations like the UK, where mental health services are stretched with limited funding. Comorbid psychiatric disorders are challenging as they require an integrated approach, with care being resource-intensive. This contributes to high costs for mental health services that may not be well-equipped to deliver integrated care for addiction and mental health problems [35].

Risks to public health have also been reported. Impulsive behaviors are common in both BD and SUD and may endanger the general public. Risky behaviors and impulsivity are common in individuals with both BD and AUD [36]. Therefore, effective risk reduction strategies are essential to protect dual-diagnosis patients and the general public. 

Pathophysiology and etiology 

BD and SUDs are complex conditions with multifactorial etiologies. Understanding the pathophysiology and etiology of these disorders is crucial for developing effective treatment strategies. 

Genetic Factors and Neurobiological Factors Contributing to Both Disorders

Genetic and neurobiological factors play a significant role in the comorbidity of BD and SUD. Research suggests that shared genetic vulnerabilities contribute to both conditions, with certain genetic variations influencing the risk of developing both disorders. For example, studies have highlighted that a common genetic factor, particularly in dopamine regulation, may increase susceptibility to both disorders [37-40].

Family studies support a strong hereditary component in BD, with first-degree relatives of affected individuals exhibiting a significantly higher risk of developing the disorder [38]. Similarly, susceptibility to SUDs has been linked to several genetic polymorphisms that affect serotonergic (HTR2A), GABAergic (GABRA2), and drug-metabolizing systems (e.g., CYP450 enzymes) [39]. These genes regulate neurotransmitter systems related to mood regulation, addiction, and behavioral response [39]. 

Taken together, these findings suggest that the frequent co-occurrence of BD and SUDs may be partially explained by shared neurobiological substrates and inherited vulnerabilities.

Neurobiological Factors

Both BD and SUDs are linked to brain regions such as the frontal lobe, cingulate cortex, and basal ganglia, which control emotion regulation and impulse control. In BD, disruptions in the dopaminergic system and neurotransmitters like serotonin and glutamate are prominent [40]. In SUDs, alterations in reward system pathways lead to dysregulated behavior related to substance use [41].

Genetic-neurobiological interactions: In dual diagnosis, overlapping brain circuits and neurotransmitter systems, such as dopamine and glutamate, may exacerbate symptoms and vulnerability [42].

Psychosocial and Environmental Influences

Psychosocial and environmental factors play a crucial role in the onset and trajectory of both BD and SUDs. Stressful life events, trauma, and ACEs are well-established as significant risk factors for both conditions [43]. These experiences can lead to dysregulation in brain development and neurobiological functioning, which in turn increases vulnerability to mood disorders and substance dependence [44]. Social determinants, such as peer influence, socioeconomic status, and environmental exposure to substances, also contribute significantly to the pathogenesis of SUDs [45,46].

Individuals with BD often turn to substance use as a coping mechanism to self-medicate or manage the mood symptoms associated with their disorder, which may increase the risk of developing comorbid SUDs [47]. This maladaptive coping strategy can worsen the course of BD, as substance use exacerbates mood instability, leading to a cyclical pattern that perpetuates both conditions [46]. The intersection of these factors highlights the complex interplay between genetic predisposition, environmental stressors, and behavioral patterns, which together shape the development and chronicity of BD and SUDs [47].

Several theories aim to explain the high comorbidity between BD and SUDs, highlighting the interaction of genetic, neurobiological, and psychosocial factors.

Self-medication hypothesis: According to this theory, people with BD might turn to drugs as a coping mechanism for their mood swings, such as using alcohol to relieve depressive symptoms or enhance the euphoria of mania. However, this maladaptive coping may worsen depressive episodes in BD [47,48].

Shared vulnerability model: This model proposes that BD and SUDs share genetic and neurobiological risk factors. Studies indicate overlapping genetic risk loci and brain abnormalities in reward processing and impulse control, contributing to the co-occurrence of both conditions [49].

Kindling and sensitization hypothesis: This theory suggests that repeated episodes of mood instability and substance use may sensitize the brain, making individuals more vulnerable to future episodes of both comorbid BD and SUDs. Repeated mood episodes in BD and substance use may also amplify cravings and mood instability [50].

Clinical manifestations and diagnosis

Subjects with BD exhibit a much higher prevalence of SUD than the rest of the population, which may be due to overlapping symptoms, misdiagnosis of mood disorders, attempts to self-medicate, or a shared genetic vulnerability [51]. Patients with dual diagnosis often present with a complex array of symptoms from both BD and SUD, which can exacerbate each other and lead to a more severe clinical presentation [52].

Symptoms and Behaviors Indicative of Dual Diagnosis

Individuals with bipolar disorder often experience a wide spectrum of symptoms, including mood swings, irritability, and insomnia, particularly during manic or hypomanic episodes, which may be further exacerbated by substance use [53]. Common manifestations include restlessness, talkativeness, impaired concentration, temper outbursts, mood lability, and overreaction to stress, making clinical differentiation from substance-induced effects challenging [53]. In childhood-onset cases, mixed episodes and rapid cycling are often accompanied by hyperactivity, impulsive behavior, severe irritability, and intense anger [53]. Additionally, symptoms such as impulsivity, poor judgment, and excessive involvement in pleasurable but risky activities are frequently observed [53]. 

Some studies suggest that temperament traits, such as sensation-seeking, are genetically determined and contribute to the overlap of symptoms between BD and AUD [54]. Distinguishing BD symptoms from those of intoxication or withdrawal is particularly challenging. Excitability or aggression could indicate mania or alcohol withdrawal, while apparent depression may be linked to either BD or cessation of cocaine use [55]. Patients with BD may abuse substances to manage their mood, relieve tension, escape reality, or boost energy. For instance, cocaine is often used to counteract the low energy of depressive phases, while alcohol, a central nervous system depressant, is frequently used to curb manic episodes [56].

**Figure 1:**
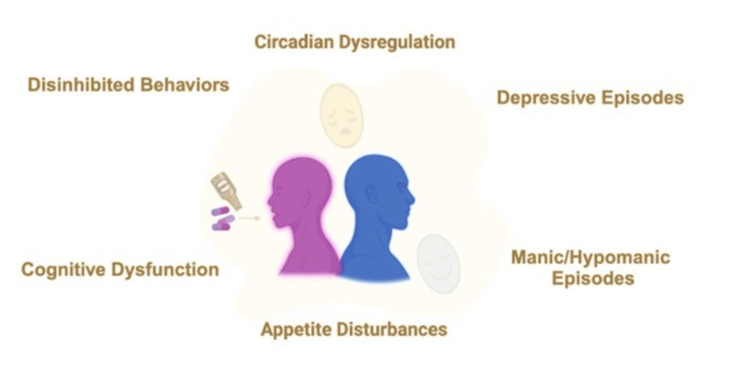
Symptoms and behaviour of comorbid bipolar disorder and substance use disorder Created by Felicia T. Bonner-Reid using BioRender.com

Conversely, some individuals may attempt to prolong the manic state with stimulants like amphetamines or cocaine, reflecting a desire to sustain manic symptoms [53-55]. This contrasts with the self-medication hypothesis, which posits that substance use is primarily an attempt to alleviate mood symptoms [53-55]. The severity of BD in patients with co-occurring substance misuse may be exacerbated by both the direct effects of substances and lower adherence to treatment regimens (Figure 1) [53].

Challenges in Differential Diagnosis

Diagnosing BD in individuals with substance abuse is particularly difficult, as the effects of prolonged drug use - such as euphoria, disinhibition, psychosis, cognitive decline, and personality changes - mimic symptoms of BD [52,53]. This often leads clinicians to attribute these symptoms to drug use without exploring the possibility of an underlying mood disorder [52,53]. Similarly, SUDs are sometimes overlooked or mistaken for secondary symptoms of a primary psychiatric disorder, with clinicians failing to recognize behaviors like "acting out" as potential indicators of BD [53].

Establishing a clear timeline for the onset of symptoms in relation to substance abuse is another challenge. It is often unclear whether BD led to substance use or vice versa. Recent evidence suggests that substance abuse is related to mood states, while others indicate that patients with SUD misuse substances regardless of their mood. The psychosocial consequences of BD may also drive secondary substance abuse [57,58]. The lack of reliable biomarkers for BD complicates diagnosis, as the affective symptoms of substance-dependent patients often resolve with prolonged abstinence and treatment, making it difficult to identify BD in individuals who are actively abusing substances [59].

During diagnostic evaluations, psychiatrists must carefully interpret the relationship between mood symptoms and substance abuse. Establishing a timeline requires an extensive history of the presenting illness, including the patient's premorbid functioning, family history, and patterns of substance use. This process often requires multiple evaluations over time, as a single assessment may not provide a clear diagnosis [60]. Dual diagnosis is associated with more severe symptoms, poorer treatment outcomes, a higher risk of relapse, and increased rates of hospitalization compared to either disorder alone [61].

Assessment Tools and Screening Methods

Several approaches can help improve diagnostic clarity in patients with dual diagnosis. These include determining the chronology of psychiatric symptom onset relative to substance use, assessing the persistence or resolution of psychiatric symptoms after intoxication or withdrawal, and evaluating the severity of psychiatric symptoms in proportion to the amount or type of substance used, as well as the duration of use [53].

Validated instruments like the Structured Clinical Interview for DSM (SCID) and the Psychiatric Research Interview for Substance and Mental Disorders (PRISM) have demonstrated high accuracy in differentiating between primary psychiatric disorders and substance-induced symptoms [62,63]. The PRISM, in particular, includes structured criteria for determining whether mood symptoms precede, coincide with, or follow substance use, enhancing diagnostic clarity in dual diagnosis populations [63]. However, despite the development of such tools, clinical interpretation remains complex, as overlapping symptomatology and patient underreporting can obscure diagnostic accuracy. This is particularly relevant in bipolar patients, where symptoms of mania or depression may mimic or be influenced by substance use.

**Figure 2:**
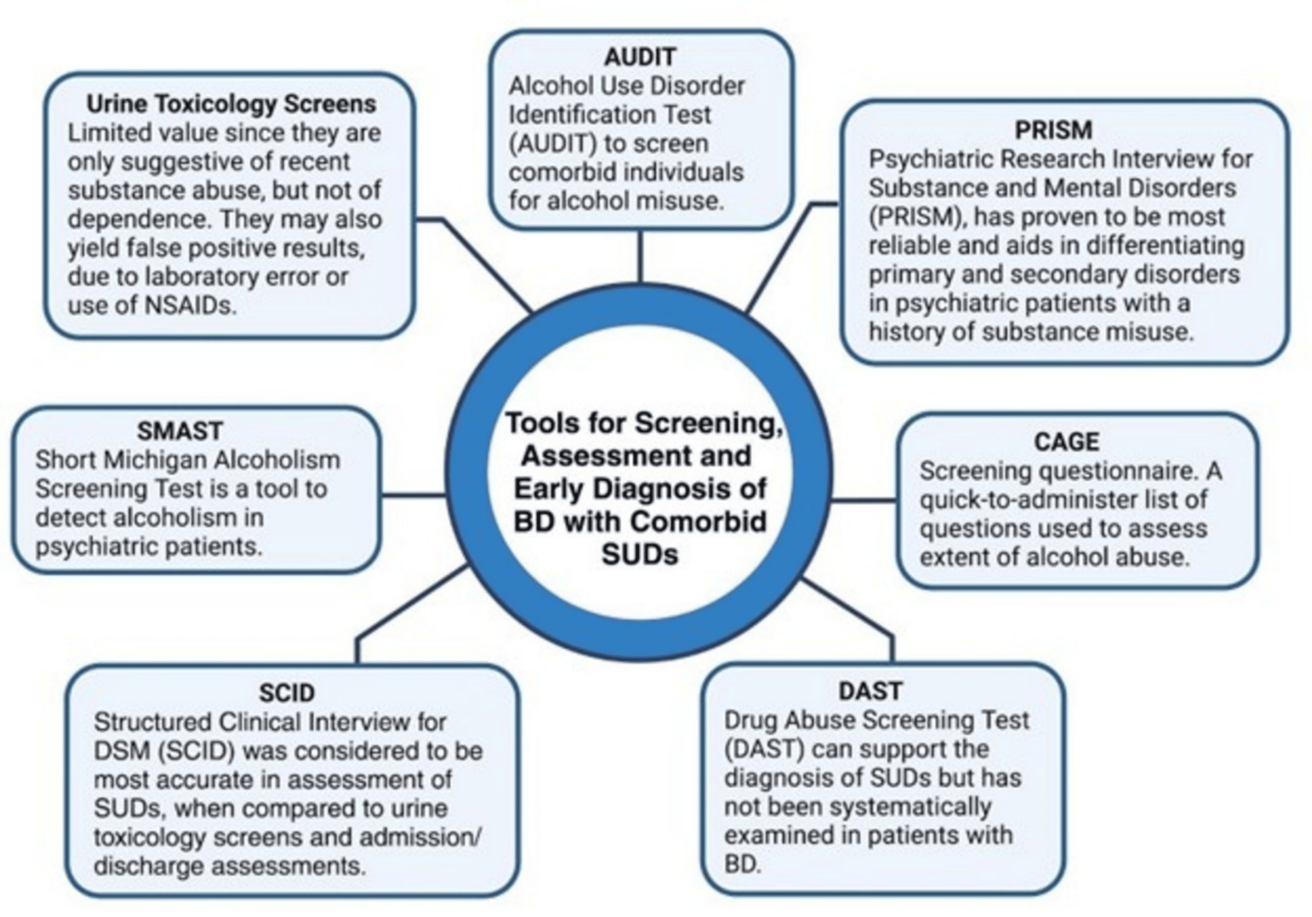
Tools for screening, assessment, and early diagnosis of BD with comorbid SUDs. Created by Felicia T. Bonner-Reid using BioRender.com from [53,55,62,63] NSAIDs: nonsteroidal anti-inflammatory drugs, SUD: substance use disorder, BD: bipolar disorder

In clinical practice, brief screening tools like the Single-Item Alcohol, Smoking, and Substance Involvement Screening Test (ASSIST) or the Alcohol Use Disorders Identification Test (AUDIT) are recommended for primary care settings due to their practicality and proven sensitivity in detecting unhealthy substance use patterns [62]. Once a patient screens positive, further assessment is advised to determine the risk level - low, moderate, or high - which informs the need for intervention or treatment referral. Routine annual screening for alcohol, tobacco, and other substances is encouraged, especially in patients on medications with potential drug interactions or those presenting with psychiatric symptoms. Nonetheless, toxicology screenings and self-reports must be interpreted cautiously, as false negatives and positives can occur. Thus, integrating structured tools with clinical judgment and patient history is essential for developing accurate, individualized care plans (Figure 2) [53,55,62,63].

Treatment approaches 

Pharmacological Interventions 

BD management is well-established with the use of mood stabilisers, antiepileptic drugs, or antipsychotics. Treatment is generally sub-classified into control of acute manic episodes and long-term management. For acute episodes of hypomania or mania, the National Institute of Clinical Excellence (NICE) CG185 guidelines in the UK recommend discontinuation of any antidepressants such as selective serotonin reuptake inhibitors, as these may precipitate manic episodes by elevating the mood [64]. The use of either a typical antipsychotic (haloperidol) or atypical antipsychotics (either olanzapine, quetiapine, or risperidone) is indicated as an initial treatment for mania.

Lithium is the gold standard for the long-term treatment of BD. Alongside mood stabilisation, it has antisuicide properties, which are particularly important in patients with a dual diagnosis who have higher suicide rates, as previously discussed [30,65]. Despite its efficacy, lithium is associated with many adverse effects, including lithium-induced diabetes insipidus and poor renal clearance due to its nephrotoxic nature [66]. Therefore, close monitoring of urea and electrolytes and overall renal function is imperative in treated patients to avoid lithium toxicity. Due to its narrow therapeutic index, careful prescribing is vital to ensure optimal bipolar control and minimisation of side effects, e.g., through brand continuity when prescribing, as products may not be bioequivalent. Thyroid disorders, particularly hypothyroidism, have also been reported with lithium via inhibition of thyroid hormone secretion [67]. An alternative is semi-sodium valproate for acute manic episodes and sodium valproate for long-term management. When used in females of childbearing age, highly effective contraception is indicated as part of the pregnancy prevention programme [68]. Valproate-based products are highly teratogenic, predisposing the exposed foetus to neurodevelopmental disorders and congenital abnormalities [69]. Recent evidence has also shown a similar risk in the offspring of males treated with valproate [70].

When comparing lithium monotherapy with semi-sodium valproate, lithium proved greater efficacy in preventing manic episodes but similar performance in preventing depressive episodes [71,72]. However, a meta-analysis found no difference in the long-term outcomes between the two agents [73].

Combination treatments using lithium or semi-sodium valproate with the dopamine agonist pramipexole have also been investigated for treatment-resistant bipolar II depression. A small-scale, double-blinded, controlled trial recruited 21 participants already taking a mood stabiliser, who were then randomised to receive either pramipexole or a placebo. The results demonstrated a statistically significant treatment effect (p < 0.03), leading to the conclusion that pramipexole was superior to placebo in reducing depressive symptoms [74]. This is an important clinical finding, as there is variable evidence regarding its efficacy in preventing depressive episodes of bipolar affective disorder (BAD), with some studies suggesting it is less effective [72,75]. Current clinical practice does not yet routinely include augmentation with pramipexole in treatment-resistant bipolar depression. Its use remains an off-label indication, typically reserved for special circumstances. However, it has shown comparable evidence to augmentation with atypical antipsychotics such as aripiprazole for the management of treatment-resistant depressive symptoms [76,77].

SUD treatment is specific to the psychoactive substance abused. Management will be explored for more commonly misused substances, namely alcohol and opiates, as they have effective pharmacological therapies available. Although cannabis was identified as a misused substance in BD, psychological interventions are widely used for this, with limited pharmacotherapeutic options [78]. However, this is an area requiring further research, given the growing non-medical use of cannabis worldwide [62]. 

In patients with AUD, treatment options to promote abstinence include disulfiram, naltrexone, and acamprosate [79]. These agents deter the individual from continuing alcohol consumption due to unpleasant side effects. For those experiencing acute withdrawal-related symptoms, a fixed-dose regimen of a long-acting benzodiazepine, chlordiazepoxide, is indicated. Intravenous thiamine (Pabrinex) may also be used to prevent or treat Wernicke’s encephalopathy, a complication of alcohol dependence [79,80]. While many pharmacological treatment options are available to AUD patients, combination treatment with psychological intervention, such as (extended) brief intervention, motivational interviewing, or cognitive behavioural therapy (CBT) may improve treatment success. The opioid antagonist naltrexone may be particularly useful in AUD patients with co-existing opioid dependence by preventing both opioid toxicity and modulating the hypothalamic-pituitary-adrenal axis to limit alcohol intake [81]. 

Similarly to AUD, opioid dependence treatment is also highly effective with methadone, a full opioid agonist, and buprenorphine, a partial opioid agonist commonly prescribed. While these agents are relatively safe, as with all CNS depressants, there is a risk of respiratory depression, particularly in patients taking other CNS depressants. This is worth considering as patients who are both opioid and alcohol dependent may be at risk of respiratory depression if excess alcohol were to be consumed while they were on treatment for opioid dependence, with either buprenorphine or methadone. 

Non-pharmacological Interventions

Role of repetitive transcranial magnetic stimulation (rTMS): rTMS is a repetitive, non-invasive technique where magnetic electrodes are attached to specific brain areas, generating impulses that stimulate dopamine production. It is claimed to reduce mental health disorders such as BD, depression, attention deficit hyperactivity disorder (ADHD), and substance misuse. This treatment is an alternative for patients who have no desire to consume pharmaceutical drugs daily. Pharmaceutical drugs tend to have high side effects and poor compliance [82]. 

The effect of rTMS on bipolar disorders: An open pilot study conducted in 2024 hypothesized that high-frequency rTMS (10 Hz) appears to be a promising intervention that significantly reduces the BD symptoms experienced by individuals. This study targeted the effect of rTMS on the dorsolateral prefrontal cortex (DLPFC). rTMS combined with high frequency (10 Hz) was used on 31 participants primarily diagnosed with BD. The treatment was delivered for six weeks with a 120% motor threshold and 3000 pulses every session. The primary outcome was measured using the Montgomery-Asberg Depression Rating Scale (MADRS), which proposed that a higher score indicates severe depression [83]. The statistically significant results of the study indicate a response rate of 87% post-treatment outcome with a lower MADRS score (p<0.001) [83]. The study supported the hypothesis that high-frequency rTMS is quite effective in providing rapid relief to patients within a shorter duration of four to five weeks. It may be a better alternative to pharmacological therapy due to its minimal side effects. However, a smaller sample size was used in the study, and so findings may not be generalisable to the wider population of BD patients. The subjects undergoing treatment in the study were also taking mood stabilizers and antipsychotic drugs, which have made the actual results of the study questionable, e.g., overestimation of positive effects of rTMS by not adjusting for co-prescribed therapies. Longer-term potential effects of using higher frequencies were also not investigated in this study [83]. 

Another single-blinded, sham-controlled study conducted in 2021 evaluated the effect of low-frequency (1 Hz) rTMS on the right dorsolateral prefrontal cortex (RDLPFC) for psychotic drug treatment-resistant patients with BD [84]. Fifty-four people experiencing severe bipolar depression were randomly assigned to the study as either active rTMS or sham rTMS. The test population consisted of the individuals allocated to the active rTMS cohort, where the treatment was delivered at 1 Hz and 110% motor threshold (MT) for three weeks while the sham group received the same stimulations but with a sham coil preventing the subject from receiving the therapeutic stimulation to minimise the placebo effect [84]. The primary outcome was measured using the MADRS score. Results were statistically insignificant as similar response rates and remission rates were reported in both experimental and control groups. Active rTMS did not outperform the sham-controlled rTMS group, further demonstrating that there was no effect on minimizing the symptoms of bipolar depression. However, the study used a limited sample size and a single-blinded design, making the study unreliable and promoting inherent bias. Insufficient dosage or frequency of 1 Hz was used, which is lower than the normal protocol. All these factors have influenced the reliable results of the study [84]. Therefore, it is unclear whether non-pharmacological therapies like rTMS are superior to pre-existing treatments and while drug toxicity is of particular concern, the established efficacy of mood stabilisers and antipsychotics makes their use justifiable and in the patient's best interests. 

Psychotherapeutic Interventions

Effective management of BD and SUD requires comprehensive psychotherapeutic interventions that address both conditions simultaneously. CBT and dialectical behavior therapy (DBT) are considered the most established and widely utilized therapies in the treatment of these disorders. These approaches have a robust evidence base supporting their efficacy in managing mood instability and substance use [85]. In contrast, twelve-step facilitation therapy (TSF), integrated cognitive behavioral therapy (ICBT), and interpersonal and social rhythm therapy (IPSRT), while promising, are less frequently implemented in clinical practice [86]. Although there is emerging evidence supporting their effectiveness, these therapies are not as widely adopted as CBT and DBT, primarily due to less extensive research and clinical experience.

CBT is a goal-oriented, time-limited therapy that focuses on identifying and changing maladaptive thoughts and behaviors. In BD, CBT aids in recognizing early mood fluctuations, establishing coping strategies, and ensuring adherence to treatment plans, thus reducing the frequency and severity of mood episodes [87]. When adapted for SUDs (CBT-SUD), the therapy emphasizes identifying triggers for substance use, developing coping mechanisms, and addressing cognitive distortions that sustain substance use patterns. CBT-SUD focuses on relapse prevention, preparing individuals for high-risk situations [88]. ICBT combines these strategies to manage both mood symptoms and substance use, integrating psychoeducation, skills training for emotional regulation, and cognitive restructuring [89,90].

An additional therapy considered reliable for the treatment of these two disorders is TSF. TSF is a structured, manualized treatment designed to encourage individuals to engage in the 12-step process, emphasizing abstinence, personal accountability, and peer support. A study demonstrated that both ICBT and TSF reduced depression and substance use during treatment. However, ICBT led to more stable long-term outcomes. While the TSF group showed a decline in depression during treatment, followed by an increase in depression afterwards. Additionally, ICBT was more effective in maintaining reduced substance use six months after treatment [90].

DBT, originally developed for borderline personality disorder, incorporates mindfulness techniques, emotional regulation, and interpersonal effectiveness skills to address the severe emotional dysregulation observed in BD and SUDs. DBT-SUD specifically targets behaviors and cognitive patterns linked to substance abuse, using strategies like "dialectical abstinence" to encourage clients to tolerate distress without substance use and to enhance emotional control [91,92].

IPSRT is a behavioral treatment that aims to help patients understand the link between their mood fluctuations and changes in their interpersonal relationships. This treatment relies on a cooperative approach, where the patient engages in structured interventions, with psychoeducation being a significant component delivered by the therapist [93]. The primary goal of IPSRT is to promote circadian rhythm stability by establishing consistent daily routines, including sleep patterns, and addressing interpersonal challenges. The patient and therapist collaborate to integrate strategies for managing daily activities into the patient’s life. Research has shown that IPSRT is more effective in preventing relapse, enhancing relationship functioning, and improving life satisfaction compared to medication management alone [94]. Although there is no direct research on the use of IPSRT with bipolar patients who also have substance misuse issues, it is expected to be beneficial, as reductions in psychiatric symptoms have been linked to decreased substance use [93,95].

These therapeutic approaches are crucial for improving outcomes in individuals with BD and SUDs, as they target the complex interplay between emotional dysregulation and substance use.

Integrated Treatment Models

Integrated treatment models are an evidence-based approach aimed at combining substance misuse and psychiatric services. It involves a multidisciplinary team of clinicians, psychotherapists, counsellors, and case managers. The aim is to facilitate the simultaneous treatment of both disorders and is thought to improve the patient's quality of life. Individualised care plans are made for patients based on resource availability, preference, and severity of each disorder [96]. When managing patients with co-existing substance misuse and SMIs, a holistic approach to care planning is essential. This requires pharmacological, psychological, and social interventions. While some studies have shown improved psychiatric symptom control with integrated treatment approaches, inadequate evidence was available to support improvement in substance misuse [97]. One study commented on the level of training received by clinicians delivering integrated treatment and whether this was adequate, as clinician knowledge and attitudes when working with SUD clients remained unchanged [98]. Therefore, it may be argued that better clinician training may improve outcomes, particularly when treating the substance misuse component. 

Prognosis and outcomes

The success of treatment for individuals with dual diagnoses of BD and SUD is influenced by several factors, including accurate diagnosis, integrated treatment approaches, and patient adherence. Misdiagnosis is common due to overlapping symptoms between BD and other psychiatric disorders, often delaying appropriate treatment and leading to poorer outcomes [54]. Treatment tailored to both disorders simultaneously is crucial; integrated programs are more effective than treating BD and SUD separately [99]. Psychosocial interventions can reduce substance abuse, while pharmacological options such as valproate and naltrexone may reduce substance use and enhance cognitive function [100].

Non-adherence is a significant barrier to successful treatment in dual diagnosis patients. High relapse rates are often linked to non-compliance with treatment regimens, and this challenge is further compounded by the complexity of treating two co-occurring conditions [101,102]. Research highlights that adherence is essential for sustained recovery, and non-adherence frequently results in relapse, hospitalizations, and increased suicide risk [103].

The long-term outcomes for individuals with both BD and SUD are generally poorer than for those with BD alone. The presence of SUD complicates treatment and is associated with increased hospitalizations, higher relapse rates, and a greater risk of suicide [104,105]. Rapid cycling, mixed episodes, and treatment resistance are particularly prevalent in patients with coexisting BD and SUD [106]. Additionally, they often exhibit greater cognitive impairments, particularly executive dysfunction, and lower psychosocial functioning [107]. These individuals face a heightened risk of poorer overall life satisfaction and impaired social and occupational recovery [108].

Future directions and recommendations 

SUDs are disproportionately prevalent among patients with BD, with higher rates of alcohol and illicit substance use compared to the general population [51,58]. Genetic vulnerability may play a role in the development of both BD and SUD, warranting further research into genetic similarities [38,55,109]. Additionally, studies are needed to examine whether the age of BD onset influences the likelihood of substance abuse. Although integrated treatment approaches combining pharmacotherapy and psychosocial interventions have shown promise, they have been insufficiently tested in controlled trials. There is also a need for more research into psychosocial treatments, particularly those that target drug cravings and incorporate motivational techniques. To improve treatment outcomes, clinicians should use standardized screening tools and adopt multidisciplinary approaches, ensuring that team members are trained to address both psychiatric and substance use symptoms. This comprehensive approach, alongside longer treatment engagement, may enhance recovery and long-term abstinence in this challenging patient population.

## Conclusions

Dual diagnosis, where BD coexists with SUD, represents a significant public health challenge due to its high prevalence and association with poor outcomes. Patients with this comorbidity face increased risks of non-adherence, psychiatric hospitalizations, rapid-cycling, mixed episodes, and higher rates of violence and suicidality. The relationship between BD and SUD is complex, as both conditions often manifest in adolescence or early adulthood, making it difficult to determine which disorder occurs first. Effective treatment for this population requires a comprehensive, multidisciplinary approach that integrates pharmacotherapy with psychosocial interventions and therapies like integrated group therapy (IGT), which addresses both disorders simultaneously. Additional investigation must be conducted to obtain an improved understanding of the mechanisms behind the high co-occurrence of BD and SUD and to develop more targeted treatment strategies, particularly for those misusing illicit substances.
